# A brief report on Primary Care Service Area catchment geographies in New South Wales Australia

**DOI:** 10.1186/1476-072X-13-38

**Published:** 2014-10-07

**Authors:** Soumya Mazumdar, Xiaoqi Feng, Paul Konings, Ian McRae, Federico Girosi

**Affiliations:** Australian Primary Health Care Research Institute, Australian National University, Building 63 Cnr Mills and Eggleston Rds, Canberra, ACT 0200 Australia; Centre for Health Research, University of Western Sydney, Penrith, NSW 2751 Australia

**Keywords:** GIS, Primary care, Primary care service area, Australia

## Abstract

**Background:**

To develop a method to use survey data to establish catchment areas of primary care or Primary Care Service Areas. Primary Care Service Areas are small areas, the majority of patients resident in which obtain their primary care services from within the geography.

**Methods:**

The data are from a large health survey (n =267,153, year 2006–2009) linked to General Practitioner service use data (year 2002–2010) from New South Wales, Australia. Our methods broadly follow those used previously by researchers in the United States of America and Switzerland, with significant modifications to improve robustness. This algorithm allocates post code areas to Primary Care Service Areas that receive the plurality of patient visits from the post code area.

**Results:**

Consistent with international findings the median Localization Index or the median percentage of patients that obtain their primary care from within a Primary Care Service Area is 55% with localization increasing with rurality.

**Conclusions:**

With the additional methodological refinements in this study, Australian Primary Care Service Areas have great potential to be of value to policymakers and researchers.

**Electronic supplementary material:**

The online version of this article (doi:10.1186/1476-072X-13-38) contains supplementary material, which is available to authorized users.

## Background

Primary Care (PC) is fundamental to an effective health system [1, 2], and is a core component of the Australian healthcare system. Over 80% of Australians consult a PC provider (most commonly a General Practitioner, GP) every year, and the Australian Primary Health Care system has been assessed as being one of the better performing among its peer nations [2–4]. The importance of locally targeted PC is well recognized across health care systems: Primary Care Trusts in the United -Kingdom were local bodies entrusted with coordinating and commissioning services; in Australia Divisions of General Practice, Primary Care Partnerships in Victoria and more recently Medicare Locals were created (and subsequently revised by a different government) to facilitate better planning of primary health care services in communities across Australia. One of the requirements of a Medicare Local was that its geography reflect local health needs and “natural catchments” [5]. While what constitutes a “natural catchment” is debatable, one approach to distinguishing catchments is through the patterns of observed PC utilization. Catchment geographies that reflect *observed* patterns of utilization of health services or patient flows are perhaps a more “natural” representation of the geographical extent of health services use within small areas as opposed to *modelled* catchment geographies such as those based on calculated travel time [6] and socio-demographic information [7]. In the geographic access literature measures of “realized access” are associated with natural catchments while measures of “potential access” relate to modelled catchments [8].

Health researchers and planners are interested in analysing PC sensitive outcomes at catchment geographies that contain the resources related to these outcomes. If the majority of patients in a geography visit healthcare providers within the geography then the targeting of appropriate policies and interventions to these geographies are more likely to be successful than if the majority patronize providers outside the geography. Finally, flows of patients to and from natural catchments, and the distances associated with these flows could help identify patterns of geographic access, and potentially areas of undersupply of services.

In spite of these advantages of natural catchment geographies they are not commonly used by policy makers and planners primarily because their construction requires substantial data and technical resources. Geographies used in mapping health outcomes and to target policies in Australia often reflect historical administrative boundaries which were not designed for the purpose of monitoring health outcomes, delivering health services or targeting health policies. For example, Medicare Locals have been used to map various health outcomes and use of health services [9] in the state of New South Wales (NSW) and elsewhere [9]. Medicare Locals were not purpose built to reflect natural catchments [5]. “Local Government Areas” (LGAs) have been used by the NSW Department of Health to map variations in various health outcomes [10]. While some public health functions are provided at a Local Government Area level [11], most services are provided either privately or by levels of government other than Local Government, and the areas are not designed for the delivery or analysis of health services. Finally, census geographies such as Statistical Areas Level 3 which were also recently used to report health outcomes and health service usage, while appropriate for reporting census information are not specifically designed for reporting health outcomes or targeting health policies [9].

In the United States [12] and Switzerland [13] where natural catchments of PC from data on observed flows of patients to PC providers have been built, these geographies have been utilized extensively by researchers [13–15]. In this brief report we describe methods to develop natural catchment areas of PC in the state of New South Wales (NSW), Australia using a large survey of people aged 45 and above representing approximately 10% of the people in NSW in that age group. Individuals aged 45 years and older comprise a significant proportion of PC users in Australia. For example, of all people that saw a GP in 2009, more than half were 45 and older [16]. We refer to natural catchment small areas of PC as “Primary Care Service Areas” (PCSAs) to be consistent with the existing US and Swiss nomenclature. While our methods are generally similar to those followed by Goodman et al. [12], there are two major differences. First, our data consists of linked large scale survey and administrative data. Second, we develop a more robust approach to managing potential instability in PCSAs, which could arise where small changes in the pattern of patient flows may cause the geographical structure of PCSAs to change.

## Methods

### Data

The dataset consists of patient survey data linked to administrative data on GP patronage. The patient data are from the Sax Institute 45 and Up Study, a survey of individuals aged 45 years and older in NSW comprising 10.9% of individuals in this age group or 267,153 individuals [17]. The survey oversamples from rural and remote areas to ensure geographic representativeness. The survey was administered across the period 2006 to 2009. A number of individual level variables are available from this survey, including the postcode of each patient’s residence. The administrative data are from the Medicare Benefits Schedule (MBS), Australia’s national government funded health insurance scheme, which subsidizes almost all private medical services in Australia. The MBS program and related data are administered by the Department of Human Services (DHS). The MBS, therefore, holds a data base which among other things includes data on all private GP consultations in Australia and which further includes information such as postcode of GP and type of consultation. For this study following Goodman [12] only those MBS items that related to GP consultations and preventative activities were used. Additional file 1: Appendix 1 lists the MBS items used in this project.

For each patient the survey data were linked to MBS data on GP consultations between 2003 and 2012. The linkage was performed by the Sax Institute using a unique identifier provided by the DHS. The methods used by Sax Institute to link data ensures the privacy of the data through well established protocols that ensures separation of identifiable data from the non-identifiable and role separation for individuals involved in the data linkage process to prevent accidental or intentional re-identification [18]. To maximize validity of the survey information for this study, MBS data for each patient were subset to a time window immediately neighboring the exact date at which the survey was administered to the patient. Three datasets, corresponding to three different time windows were created: complete time period 2003 – 2012; one year period: 183 days (half year) before and 183 days after the 45 and Up Study survey date of participants; two year period: 365 days (one year) before and 365 days after the survey date of participants. After comparisons between the three datasets, the one year window dataset was considered optimal (Additional file 1: Appendix 2), resulting in a dataset of 255,461 patients. Note that a patient may make multiple visits to one or more GPs. This study and the 45 and Up Survey were approved by relevant ethics committees.

### Pre processing of data

The data for the construction of a PCSA are organized in an origin–destination (O-D) matrix that has patient postcodes on the rows and provider postcodes on the columns. Each cell carries information about the strength of the flow of patients from a patient postcode to a provider postcode. PCSAs are expected to represent natural travel behavior of patients and it is possible for people with chronic illnesses who make more than the average number of visits to a GP to bias the geography. To minimize this bias, each patient is assigned one “vote” which is split in proportion to the number of visits made by the patient to a postcode [12]. The final product is thus a spatial interaction matrix or origin–destination (O-D) matrix of total votes flowing between patient and provider postcodes. Each cell in the matrix represents the total number of votes moving from a patient postcode to a provider postcode.

The above matrix locates patients and provider GPs in postcodes. As in some other jurisdictions, Australian postcodes are not designed to have coherent spatial geographies. Since, PCSAs are spatial entities, Postal Areas (POAs) which are spatial approximations of postcodes developed by the Australian Bureau of Statistics (ABS) are used in this project. A total of 621 ABS-2011 POAs exist in NSW with a median resident population of ~3,500 and a median of 243 people aged 45 or over in the study sample.

However POAs suffer from two problems. Some postcodes are aspatial entities such as locked bags (Post Office Boxes) in Post Offices with no corresponding POA. A total of 21 of these were found in our data and assigned an approximate POA. Second, 35 POAs were found to be geographically split, with two or more areas with the same POA number physically separated by other POAs. Split POAs arise from split postcodes.To resolve this problem each non-contiguous section of the POA (along with all other POAs) was given a new identifying code - we call these new geographies as gPOAs. A total of 99 gPOAs geometries were created from the 35 split POAs. The patient provider O-D matrix was modified by weighting the total number of patients and votes from each split POA by the proportion of total Usual Resident Population (URP) in the split POA. If a provider GP POA was split, then incoming numbers of votes were also split in proportion to the URP at the new geometries. A total of 884 gPOAs were assigned to the postcodes in the O-D matrix.

Some survey respondents who reside in the border areas of NSW obtain the majority of their GP services outside NSW. For example, many of the patients living in the far South-Western Regions of NSW obtain their services from the town of Mildura just across the NSW border in the state of Victoria. To account for the provider usage patterns of these and similar patients, a buffer of 223 POAs around NSW was also also assigned to the destination postcodes in the O-D matrix.

### Allocation procedure

Patient gPOAs are assigned to provider gPOAs using a “marginal rule” [19] used previously by Goodman [12]. Each patient gPOA is assigned to the provider gPOA that receives the maximum percentage of votes from the gPOA (which will of course frequently be the patient gPOA itself). The geographical aggregate of assigned patient gPOAs together with the provider gPOA form a PCSA.

A source of instability in the allocation procedure arises when two provider gPOAs are “tied” with the same number of votes from a patient gPOA. Goodman et al. [12] manage exact ties by attaching the patient gPOA to the nearest of these provider gPOAs. However, when votes are approximately equal, small changes in the number of votes can change an assignment between areas. This situation is most likely to arise when there are only a small number of patients in a gPOA, an effect which is magnified in this study due to the use of a population sample. Goodman considered geographies with greater than 140 patients as stable, but the method behind this choice was not published. To identify situations of instability we create confidence intervals around the fraction of votes from each patient gPOA going to each potential provider gPOA, and declared “ties” if the percent of votes for the highest and second highest percent set of votes were not significantly different one from another (Details on the construction of Confidence Intervals are in Additional file 1: Appendix 3). This accommodated the issue of small numbers without requiring a size cut-off, and also means “near ties”, for example, 49% vs 51% may be treated as ties even in larger gPOAs. A total of 151 such “ties” were identified and allocated to the nearer of the two gPOAs. Of course, in the case of a tie or near tie the reallocation will frequently be to the gPOA originally selected (this will happen one time in two with pairwise ties).

### A posteriori processing of PCSAs

When the above procedure resulted in a patient gPOA being assigned to a non-contiguous PCSA, gPOAs were reassigned using the allocation procedure described in the following paragraph, which relies on the notion of the Localization Index (LI) [1, 13]. This index is the percentage of votes of patients living within a PCSA that go to GPs in the PCSA. Since by definition PCSAs should have high LIs, PCSAs with low LIs present a problem. Following Goodman et al.’s version 3 PCSA algorithm [20], we identify PCSAs with LIs less than 10 (i.e. less than 10% of the votes go to the PCSA itself) and reassign their constituent gPOAs to other PCSAs using the usual assignment rules. PCSAs with LIs between 10 and 30 were reassigned if the number of votes it sends to itself is less than 67% of the votes it sends to any other PCSA.

### Reallocation procedure

The reallocation procedure for non-contiguity or low LIs is as follows [20]. The first choice for reassignment was to a contiguous PCSA which among all contiguous PCSAs received the highest percentage of votes from the patient gPOA. If only one contiguous PCSA received votes from the patient gPOA then this PCSA received the assignment. If no contiguous PCSA received any votes from the patient gPOA then the gPOA was reassigned to the closest contiguous PCSA. If more than one contiguous PCSA received votes from the patient gPOA then the first and second gPOAs were tested for ties or to assess if proportions of votes were significantly different – if different the first choice was retained, if not the nearest contiguous PCSA of the ties or near ties was chosen.

**Figure 1:**
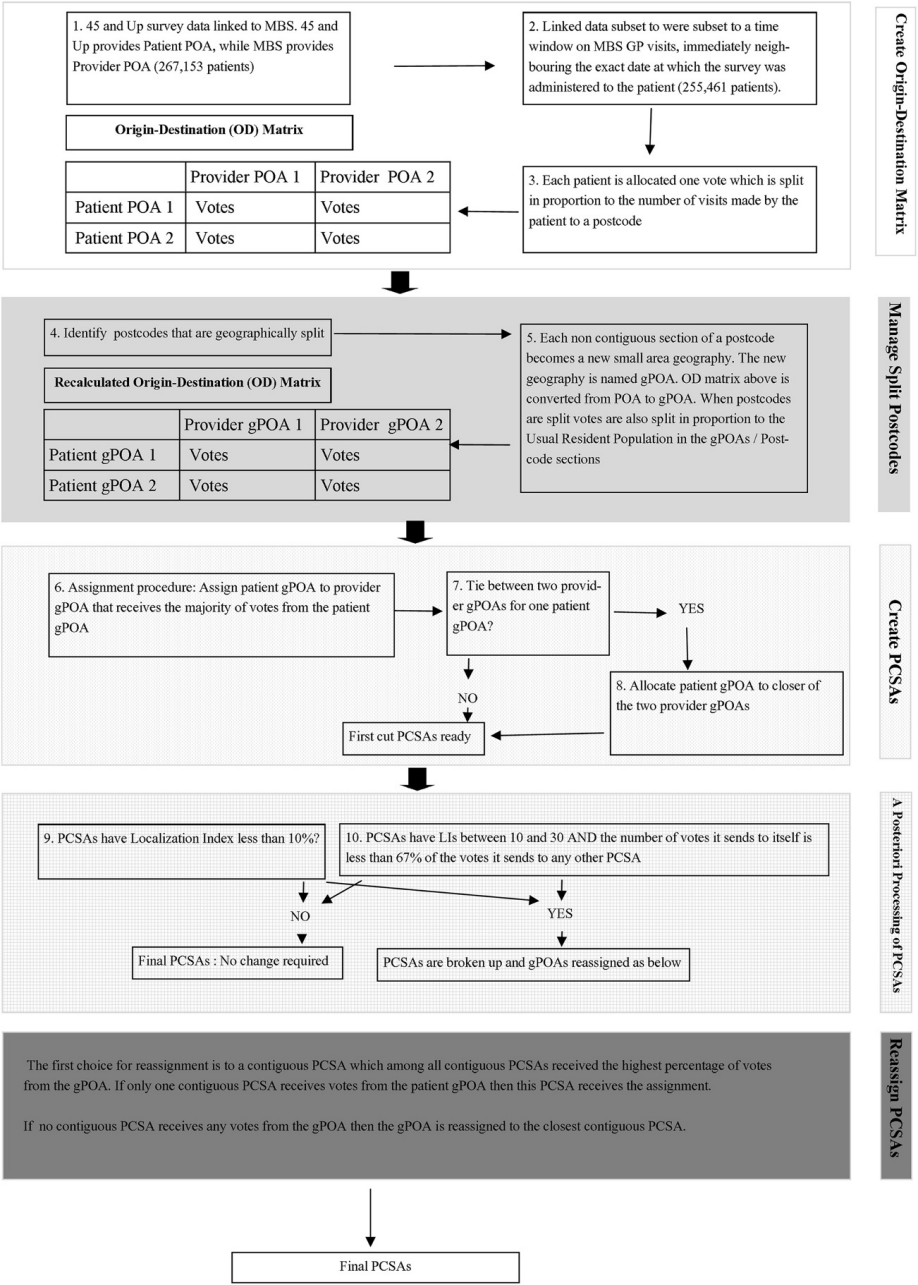
**PCSA creation flow diagram.**

Figure 1 summarizes the above PCSA creation method using a flow diagram. Resulting PCSAs were attached to Australian geographic rurality classification data from ABS that ranks POAs from Metropolitan to Very Remote over an increasing gradient of rurality [21].

## Results

**Figure 2:**
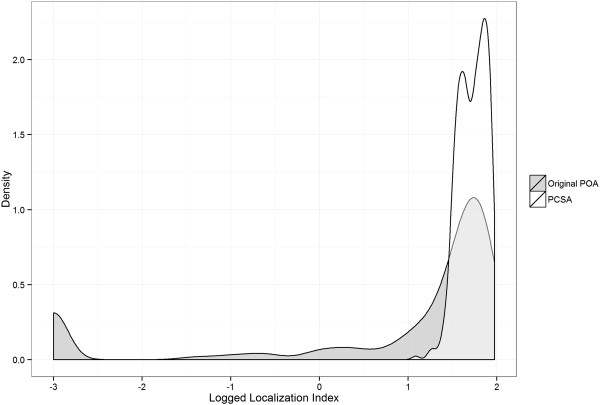
**The distribution of Localization Indices for PCSAs show higher values for PCSAs.**

**Figure 3:**
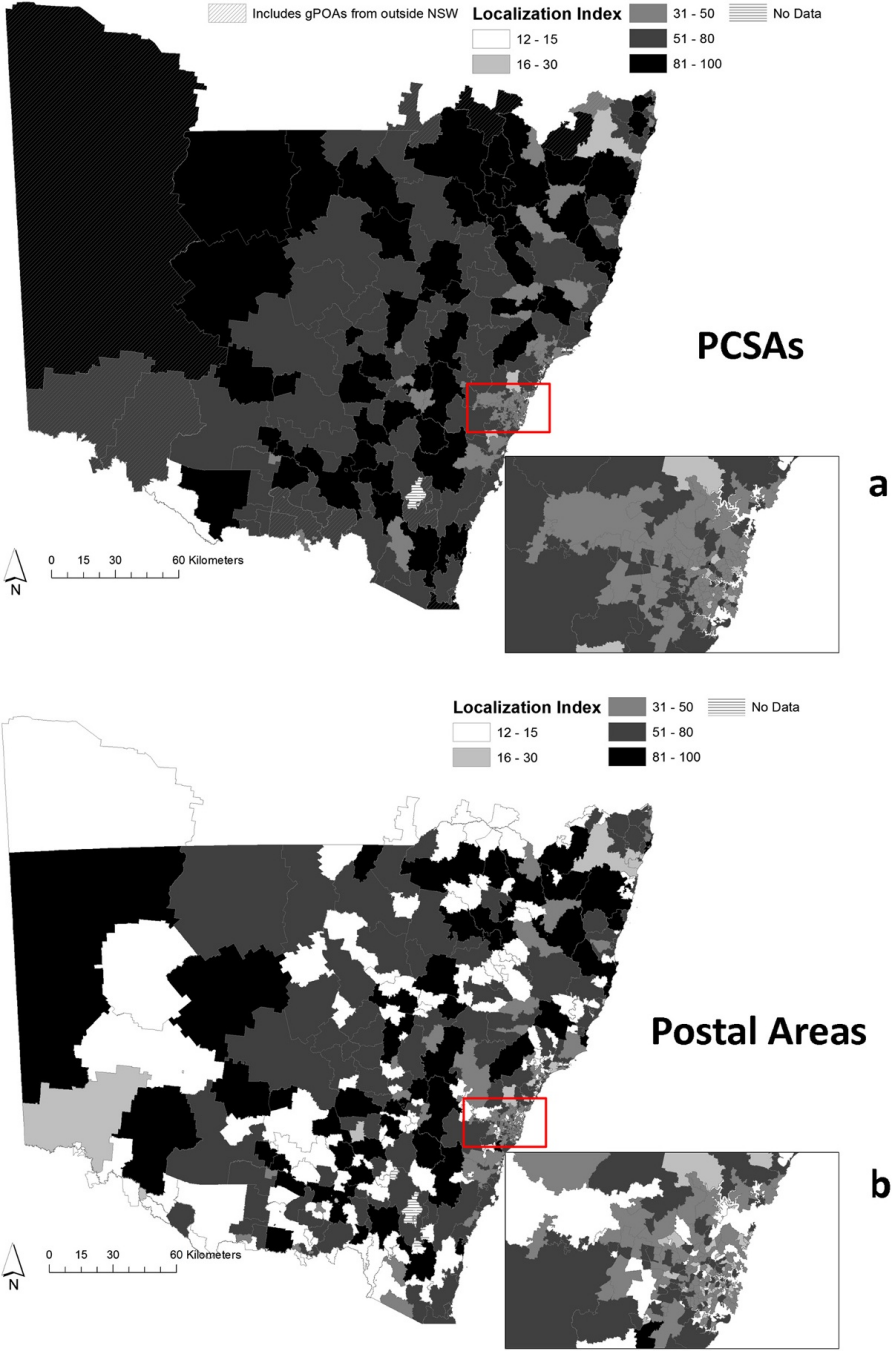
**PCSAs have higher localization than POAs.** Panel **a)** Localization at PCSAs Panel **b)** Localization at Postal Areas. The insets display Localization Indices in Sydney.

A total of 392 PCSAs were generated from 884 gPOAs. Figure 2 displays a histogram of Localization Indices of the PCSAs compared to those in the original patient gPOAs. Figure 3 displays maps of LIs at PCSAs and LIs at gPOAs from which the PCSAs were created. While, the median LI in gPOAs with patients is 38%, the comparable statistic for PCSAs is 55%.While the LIs became smaller as gPOAs became more remote, they became larger as PCSAs became more remote (Table 1). Remote Australian PCSAs are larger in area (mean 19,776 sq. km) compared to metropolitan PCSAs (mean 117 sq. km) reflecting the larger size of rural and remote Australian POAs. The median number of patients in a PCSA was 394. The correlation between median number of patients and LI Index at PCSAs is 0.23. A total of 15 PCSAs included gPOAs from outside NSW, and were identified as border PCSAs. Eighty two percent of the PCSAs were composed of 2 or less gPOAs.

**Table 1:** **Localization increases with increasing rurality in PCSAs while rural Postal Areas have lower Localization Indices**

Remoteness Classification	Mean Localization Index at PCSAs	Mean Localization Index at gPOAs
Major Cities	46	37
Inner Regional	62	37
Outer Regional	70	34
Remote	77	20
Very Remote	83	6

## Discussion

We offer an approach that attempts to appropriately manage the sources of instability in creating PCSAs while trading this robustness for the risk of attributing more gPOAs to their nearest neighbour than would otherwise be the case. This is relevant not only in studies using sample data, but also in the census data context for example, complete US Medicare data. We identify patient postcodes at most risk of being differently allocated by small changes in aggregate patient flows, and use a conservative allocation procedure to assign them to neighbours. While some postcodes may be wrongly assigned the overall impact is likely to be small since these postcodes usually have a small number of patients.

We found the same level of localization in PCSAs as in the USA [12] and Switzerland [13]. This consistency may be the reflective of certain universalities in the patterns of patient mobility, and should be subject to further research. Another finding consistent with US PCSAs is that localization increases with rurality, a finding which while being intuitive finds further empirical validation from this study.

Geographic variations in healthcare costs and use have been brought into the spotlight by the recent debate in the United States on whether Medicare related incentive costs should be targeted geographically [22–24]. After a protracted debate a general conclusion was reached (summarized by the Institute of Medicine Reports [23, 24]) that geographic targeting of incentives in the context of tertiary care is not the best approach and better methodologies are required. At no point during the debate however, was the validity of the methodologies that were used to create Hospital Service Areas or Primary Care Service Areas (as opposed to the methods used to analyse data using these geographies) brought to question underscoring the solid conceptual and methodological foundations on which these geographies stand. Indeed, the Health Resources and Services Administration, an agency of the US Department of Health and Human Services states “Primary Care Service Areas (PCSAs) define service areas across the U.S. and are a useful tool for analyzing the distribution of health professionals, primary care services and access to primary care.” Thus it is not surprising that PCSAs are continually being used to study primary care relevant issues in the US [14, 15].”

We use a survey of people 45 and older linked to administrative data to create PCSAs. Since these data are not a census of the active patient population, there is always the possibility of bias (our smallest PCSA represents a population of at least 250 people 45 and over). While the survey represents 10% of the people in NSW 45 and older and around 4% of the NSW population, of all people that saw a GP in 2009 more than half were 45 and older [16]. Nevertheless, bias may arise if the geographical patterns of GP patronage of the sample population are not representative of the usage patterns of the total population in a given area. In the United States PCSAs were created from Medicare-US data, representing a population of users 65 and older and validated against other datasets [12], such datasets however, are exceedingly difficult to obtain in Australia due to privacy legislations in Australia [25, 26]. Nevertheless, the LIs of the PCSAs created in this study are in agreement with what was found in Switzerland and US, which underscores their reliability in spite of being created from a much smaller numerical base than the US and Swiss PCSAs.

While we use a maximal allocation method for building these PCSAs following an existing, validated method with a large literature on research using PCSAs created using these methods, newer methods of optimization based regionalization techniques automate some of the ad hoc parameters used in this method [19, 27]. Utilization of these methods remains a possible avenue of future research.

## Conclusion

Patients in New South Wales for many reasons will travel to obtain GP care. As a result, any studies of utilisation of PC or attempts to measure PC workforce shortage need to be designed with an understanding of the relationship between the area where a patient lives and the area where they receive most of their PC. This paper identifies PCSAs for NSW and is the first such geography for Australia, using a tried and tested methodology with improvements for enhanced robustness.

### Consent

This research utilizes secondary data, thus individual consent was not required.

## Endnote

^a^Goodman et al. have used the term “Preference Index”. They also used fractions while we use percentages to represent this index.

## Electronic supplementary material

Additional file 1: Appendix 1: MBS Items used for PCSA creation, **Appendix 2.** Comparing PCSAs created from different datasets, **Appendix 3.** Defining ties. (DOCX 66 KB)

## Abbreviations

ABS: Australian Bureau of Statistics
DHS: Department of Human Services
GP: General Practitioner
LI: Localization Index
MBS: Medicare Benefits Schedule
NSW: New South Wales
O-D: Origin–destination
POA: Postal Area
gPOA: Postal Areas (modified Postal Areas created for this project)
PC: Primary Care
PCSA: Primary Care Service Area
US: United States.
