# Supplementary material for: A brief report on Primary Care Service Area catchment geographies in New South Wales Australia
Source: Int J Health Geogr. 2014 Oct 7;13:38. doi: 10.1186/1476-072X-13-38 (PMC4197238; doi:10.1186/1476-072X-13-38)
Supplement: Supplementary file 1 — Additional file 1: Appendix 1: MBS Items used for PCSA creation, Appendix 2. Comparing PCSAs created from different datasets, Appendix 3. Defining ties. (DOCX 66 KB) [file 12942_2014_605_MOESM1_ESM.docx]

**Appendix 1: MBS Items used for PCSA creation:**

| MBS Item Name | MBS Item Number | MBS Item Description |
| --- | --- | --- |
| A1-GP attendances | 3,23,36,44 | CONSULTATION AT CONSULTING ROOMS |
| A2-MP other than GP | 52,53,54,57 | CONSULTATION AT A CONSULTING ROOM |
| A7 Acupuncture** | 173, 193, 197, 199 | Place other than a hospital |
| A11 Urgent after hours attendances** | 597,598,599,600 | CONSULTATION AT A CONSULTING ROOM |
| A14 Health assessments** | 701, 703, 705, 707, 715 | Need not be in rooms but usually will be |
| A15-GPMP | 721,723,729, 732 | CONSULTATION AT A CONSULTING ROOM |
| A18-GP,  cervical screening | 2497, 2501, 2503, 2504, 2507 | CONSULTATION AT CONSULTING ROOMS |
| A18- GP asthma | 2546, 2552, 2558 | CONSULTATION AT CONSULTING ROOMS (cycle of care) |
| A18- GP diabetes | 2517, 2521, 2525 | CONSULTATION AT CONSULTING ROOMS (cycle of care) |
| A19-non referred cervical screening | 2598, 2600, 2603, 2606 | Professional attendance at consulting rooms |
| A19- non referred, asthma | 2664, 2666, 2668 | CONSULTATION AT CONSULTING ROOMS |
| A19-non referred, diabetes | 2620, 2622, 2624 | CONSULTATION AT CONSULTING ROOMS |
| A20-GP mental health care plans | 2700, 2701, 2712, 2713,2715, 2717, 2721, 2725 | CONSULTATION AT CONSULTING ROOMS |
| A22-after hours (GP attendances) | 5000, 5020, 5040, 5060 | CONSULTATION AT A CONSULTING ROOM |
| A23- after hours (non-referred) | 5200, 5203, 5207, 5208, | CONSULTATION AT A CONSULTING ROOM |

**Appendix 2: Comparing PCSAs created from different datasets:**

Three datasets, corresponding to three different time window sizes or time ranges were created:

- The full time period 2003 – 2012 for which MBS data were provided (Called Full time period data)
- One year period: 183 days (half year) before and after the 45 and Up Study survey date of participants (Called Halfyear data)
- Two year period: 365 days (one year) before and after the survey date of participants (Called Oneyear data)

The figure below (Figure A1) illustrates the difference between these datasets.

**Fig A2: Three sets of linked survey-administrative data**


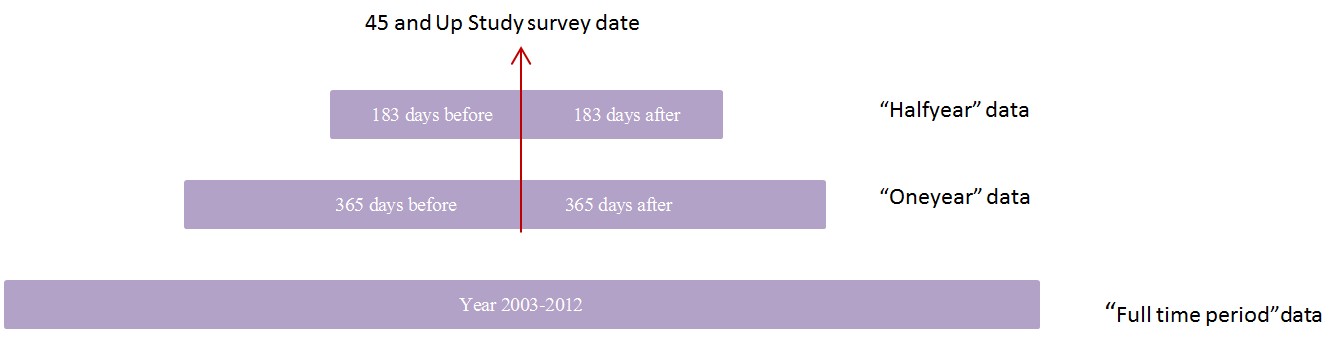


We base our choice of dataset by examining/comparing preliminary outputs (PCSAs) from each of them. We ask two questions. First, how similar or different are the Localization Indices (LIs) of PCSAs (See methods) created from the three datasets? Second, we ask, how similar or different are the geographies of the PCSAs created from the three datasets. We are specifically interested in the number of people that are differentially allocated when one dataset is chosen over another. We believe that population serves as a better metric of comparison than geometric area.

We use the population Jaccard Coefficient, a modified version of the area Jaccard Coefficient ([Farmer and Fotheringham 2011](file:///C:\Users\u5253176\AppData\Local\Temp\l)), to compare the level of commonality between the PCSAs created by using the three datasets. This is calculated as the intersection, agreement or common population between two sets of PCSAs, divided by the total patient population in any one set of PCSA. There are small differences in patient populations in gPOAs in the three datasets, thus for the purpose of this analysis; the patient population in the one year dataset was used as divisor. Using other years, does not significantly change the results. To illustrate the population Jaccard Coefficient calculation with an example, if in one PCSA dataset corresponding to one time window, PCSA X is comprised of gPOA 2111, 2112 and 2113, while in another PCSA dataset corresponding to another time window the gPOAs (2111, 2112 and 2113) comprise PCSA Y to its entirety then an agreement is achieved. However if PCSA Y were to comprise of an additional gPOA 2114, then an agreement is not achieved on gPOAs 2111, 2112, 2003 and 2114. Their patient populations would therefore not contribute patients to the numerator of the Jaccard coefficient. Table 1 compares the Localization Indices at PCSAs created using the three datasets, in addition to other information. Table 2 displays the Population Jaccard Coefficient between the One year dataset and the rest.

- Farmer, C. J. Q. and A. S. Fotheringham. 2011. “Network-based functional regions.” *Environment and Planning A* 43(11): 2723 – 41 %U <http://www.envplan.com/abstract.cgi?id=a44136>.

**Results:**

Table 1: Localization Indices of the three datasets

| **Datasets** | **Number of PCSAs obtained** | **Localization Index** | | | | **Total Count of patients in final PCSAs** |
| --- | --- | --- | --- | --- | --- | --- |
|  |  | **Mean** | **Median** | **Min** | **Max** |  |
| **Oneyear** | 393 | 56 | 55 | 12 | 100 | 255,461 |
| **Halfyear** | 397 | 57 | 55 | 19 | 100 | 245,958 |
| **Full time period** | 389 | 55 | 54 | 12 | 93 | 261,334 |

Table 2 Population Jaccard Coefficients between the One year and other datasets

|  | **One year** |
| --- | --- |
| **Half year** | 96% |
| **Full time period** | 86% |

The PCSAs created from the one year dataset are similar to the ones created from the other datasets. This dataset is both logically reasonable in terms of having likely valid locations of patient residence postcodes; and being intermediate between the very short and very long time periods. We therefore chose this dataset as optimal for publishing PCSA geographies.

**Appendix 3: Defining ties:**

Defining and validating ties:

To evaluate to which gPOA of two given provider or GP gPOAs a patient gPOA should be assigned, confidence intervals need to be constructed around the fraction of votes that the patient gPOA sends to the provider gPOAs. A robust method to do this would be to bootstrap this statistic from the data representing votes from each patient in the patient gPOA to the provider gPOAs. However, this dataset consists of millions of records and bootstrapping Confidence Intervals (CIs) would generate an immense computing load.

To simplify this problem, we first calculate CIs using a binominal approximation. Let p_ij_ be the fraction of votes, rounded to the nearest whole number, from the i’th patient gPOA to j’th provider gPOA and n_i_ be the number of patients in the patient POA. Then the 95% CI for fractions of vote is estimated as the binomial CI:

CI (p_ij_) = p_ij_ ±1.96* (p_i_ *(1- p_ij_)/n) ^0.5^

While the use of the binomial formulation would be correct if patients were allocated to provider gPOAs, votes which are frequently less than one are allocated so the binomial formulation will overstate the standard errors leading to confidence intervals that are inflated by around 50% (estimate found by simulations), and some ties are likely to be invalid.

Thus we use the above definition of ties to shortlist a set of ties, where at least two provider gPOAs are competing for the assignment of the same patient gPOA. We calculate the bootstrapped standard errors and means for the fraction of votes from patients in this paired shortlist of patient gPOAs-provider gPOAs or p_ij_’s. We then use these bootstrapped confidence intervals to trim the above list of ties. This two-step strategy of first using overstated analytical confidence intervals and then narrowing down the list using bootstraps helps avoid unnecessary intensive computation of bootstrapping confidence intervals for all combinations of patient and provider gPOAs.

Testing Tie Parameters:

There are two assumed parameters in defining the above ties. Changing these parameters may change the resulting assignments. First we may change the size of the confidence intervals (CIs) around the fraction of votes. Second, we consider the top two voting gPOAs with tied confidence intervals as an ‘instability set’, that is to say that small changes in the fraction/percent of votes from these gPOAs could change their assignment to a provider gPOA. However, it is possible that this ‘instability set’ can consist of more than two gPOAs with tied vote fractions. A larger set would imply a different gPOA from the top two voting gPOAs being assigned to a given provider gPOA, and thus a different PCSA structure. For evaluating the effect these alternatives have on the PCSAs we ask the question, - “What is the maximum of the percent patient population that would be differently assigned if the definition of ties were changed?” We test the effect of each of these two alternatives (on the one year dataset) below

1. Changing the size of the Confidence Intervals: Changing the analytical CIs from 95% to 90% causes at most 5817 (<3%) patients to be reassigned. Similarly a change from 95% to 99% causes at most 9903 (<4 %) patients to be reassigned.

2. Increasing the size of a tied set: If the tied set were expanded to three gPOAs then at most 6467 patients (<3%) would be reassigned. If the set were expanded to include the top four gPOAs then at most a further 2324 (<1%) patients will be reassigned.

Thus, the current tie definition parameter choices were considered reasonable.
